# Associations between enteric pathogen carriage and height-for-age, weight-for-age and weight-for-height in children under 5 years old in urban Dhaka, Bangladesh

**DOI:** 10.1017/S0950268820000369

**Published:** 2020-02-27

**Authors:** David Berendes, Drew Capone, Jackie Knee, David Holcomb, Sonia Sultana, Amy J. Pickering, Joe Brown

**Affiliations:** 1 Division of Foodborne, Waterborne, and Environmental Diseases, National Center for Emerging and Zoonotic Infectious Diseases, US Centers for Disease Control and Prevention, Atlanta, GA, USA; 2 School of Civil and Environmental Engineering, Georgia Institute of Technology, Atlanta, GA, USA; 3 Department of Environmental Sciences and Engineering, Gillings School of Global Public Health, University of North Carolina, Chapel Hill, NC, USA; 4 International Centre for Diarrheal Disease Research, Bangladesh (icddr,b), Dhaka, Bangladesh; 5 School of Engineering, Tufts University, Medford, MA, USA

**Keywords:** Diarrhoea, enteric infection, *Giardia*, stunting

## Abstract

Nutritional factors and infectious agents may contribute to paediatric growth deficits in low- and middle-income countries; however, the contribution of enteric pathogens is only beginning to be understood. We analysed the stool from children <5 years old from an open cohort, cluster-randomised controlled trial of a point-of-collection water chlorinator in urban Bangladesh. We compared the presence/absence of 15 enteric pathogens detected via multiplex, molecular methods in the stool with concurrent *Z*-scores/*Z*-score cut-offs (−2 standard deviations (s.d.)) for height-for-age (HAZ/stunting), weight-for-age (WAZ/underweight) and weight-for-height (WHZ/wasting), adjusted for sociodemographic and trial-related factors, and measured caregiver-reported diarrhoea. Enteric pathogen prevalence in the stool was high (88% had ≥1 enteric pathogen, most commonly *Giardia* spp. (40%), *Salmonella enterica* (33%), enterotoxigenic *E. coli* (28%) and *Shigella* spp. (27%)) while reported 7-day diarrhoea prevalence was 6%, suggesting high subclinical infection rates. Many children were stunted (26%) or underweight (24%). Adjusted models suggested *Giardia* spp. detection was associated with lower HAZ (−0.22 s.d., 95% CI −0.44 to 0.00; prevalence ratio for stunting: 1.39, 95% CI 0.94–2.06) and potentially lower WAZ. No pathogens were associated with reported diarrhoea in adjusted models. *Giardia* spp. carriage may be associated with growth faltering, but not diarrhoea, in this and similar low-income settings. Stool-based enteric pathogen detection provides a direct indication of previous exposure that may be useful as a broader endpoint of trials of environmental interventions.

## Introduction

Almost 27% of the world's preschool children are stunted (defined specifically as height-for-age *Z*-scores ⩽2 standard deviations below the mean), with hundreds of millions more underweight (low weight-for-age) or wasted (low weight-for-height), especially in low- and middle-income countries [1, 2]. Beyond the poor nutrition that characterises many diets in these settings, the role of recurrent or persistent enteric infections in undernutrition, and especially linear growth faltering (defined as any low height-for-age), is incompletely understood [3, 4]. These enteric infections may elicit inflammatory immune responses that can lead to chronic gut inflammation, and subsequent morphological changes, reduced absorptive capacity for nutrients and other sequelae [5]. This condition, termed environmental enteric dysfunction (EED), expands the scope of potential health impacts associated with enteric infections to include subclinical infections that contribute to gut inflammation without the presence of diarrhoea [6].

While the diarrhoeal disease affects more than 1.7 billion children worldwide each year [7], the burden of enteric infections – inclusive of asymptomatic infections – is even larger, especially in young children [8–10]. A multisite cohort study of both non-diarrhoeal and diarrhoeal stool from children <2 years old – when impacts of environmental conditions on linear growth, may be most important [11] – observed that more than two-thirds of the children were shedding an enteric pathogen in the stool, indicative of current or previous infection or asymptomatic carriage [9]. Notably, while about 77% of diarrhoeal stool was positive for ≥1 enteric pathogen, 65% of non-diarrhoeal stool was also positive across sites [9]. The prevalence of bacterial, viral and protozoan infections varies by region and age range [8, 9], and thus infection-related drivers of growth faltering may also vary, making contextualisation of the local burden of enteric infection important [12].

Although environmental interventions focused on reducing exposures to faecal pathogens – for example, improved water, sanitation and hygiene (WASH) infrastructure – have typically been evaluated by their effectiveness in reducing diarrhoea [13], recognition of the potential impact of subclinical infections is broadening the focus of such studies to target both symptomatic and asymptomatic enteric infections [14]. By extension, the impact of interventions on linear growth and other nutritional outcomes is being measured. Two studies have already reported significant effects of improved community-level sanitation on linear growth in the absence of measurable effects on diarrhoea [15, 16], but other major randomised-controlled trials have not observed such outcomes [17–19]. The challenges and expenses of discerning and interpreting the pathogen-specific biological and environmental pathways through which EED acts [20] have resulted in the limited analysis of pathogens and child growth. However, recently-completed multisite studies may add to these results in the near future [17, 18].

As WASH implementers strive to impact not only faecal pathogen exposures but also linear growth, it is important to understand the roles and burdens (e.g. background circulation) of specific pathogens. This importance is twofold: (1) enteric pathogens may be aetiological agents of symptomatic illness with short-term impacts; and perhaps more importantly, (2) those same enteric pathogens may also be the cause of asymptomatic infection or be commensally carried in the gut, resulting in longer-term impacts [4]. Pathogen-specific approaches are likely not the long-term solution to controlling enteric infections – many enteric pathogens share transmission pathways and may be controlled via system-level improvements in WASH infrastructure [21]. However, improved understanding of pathogen- and pathway-specific contributions to poor nutrition in low-income settings can inform intervention strategies, exposure measurement and risk assessment [22]. Within the context of a cluster-randomised controlled trial of an automated point-of-collection water chlorination device in urban Bangladesh [23], we conducted a sub-analysis exploring associations between pathogen carriage, symptomatic diarrhoea and anthropometric outcomes (height-for-age, weight-for-age and weight-for-height) among children <5 years old to identify potential pathogens of importance for these outcomes. Results from this analysis can contribute to future efforts to understand and mitigate these pathogens – regardless of symptoms – with environmental interventions and highlight the importance of subclinical infections in nutritional outcomes.

## Methods

This analysis was nested within a previously described, cluster-randomised controlled trial of a point-of-collection chlorinator on self-reported diarrhoea in children in two urban and peri-urban communities in and around Dhaka, Bangladesh (NCT02606981) [23]. While this analysis is cross-sectional for the specified outcomes in children in both intervention and control groups, the parent trial included data collection from an open cohort of children <5 years old (enrolled for a baseline (pre-intervention) evaluation between July and November 2015, and followed-up approximately every 2 months over seven post-intervention rounds (rounds 1–7) from November 2015 to December 2016). Briefly, the parent trial was conducted in two low-income neighbourhoods: Tongi, a community outside Dhaka, and Dhaka Uddan, a community within Dhaka city. Compounds of between 1 and 72 (median 5) households within these communities were randomised to intervention and control groups by water point (receiving/not receiving a passive chlorine-dosing device), stratified by site. Water points in each site were ordered by the number of children <5 years old residing in households accessing and using the enrolled water points as their primary drinking-water source, then paired in descending order to either treatment/control status via a random number generator [23]. The parent study determined the eligibility of water points by the presence of a water storage tank compatible with the dosing device, and compounds had to have ≥1 child <5 years old using that water point as their primary source of drinking water. Follow-up was discontinued if the child aged out of the cohort or had migrated out of the study area and could no longer be located. The study protocol was approved by the scientific and ethical review boards of the International Centre for Diarrheal Disease Research, Bangladesh (icddr,b, protocol 14022) and the human subjects institutional review board of the Stanford University (protocol 30456) [23]. CDC coauthors (Berendes) received de-identified data for analysis as per a non-disclosure agreement with the Principal Investigator (Pickering).

### Stool collection and analysis

Field staff collected whole stool specimens from all study children during the sixth round of follow-up by instructing caregivers to fill sterile plastic collection containers immediately after child defecation. Field staff delivered collection kits to households and attempted to retrieve the stool samples the following morning. Field staff conducted up to three follow-up visits per household to retrieve specimens. Specimens were kept at −80 °C at the International Centre for Diarrheal Disease Research, Bangladesh (icddr,b) in Dhaka, Bangladesh and transported on dry ice to the Georgia Institute of Technology (Atlanta, GA, USA) where they were stored at −80 °C until analysis.

We used the Luminex xTAG^®^ Gastrointestinal Pathogen Panel (GPP) to analyse the stool specimens. The GPP is a multiplex end-point RT-PCR assay used to determine the presence or absence of 15 enteric pathogens: *Campylobacter*; *Clostridium difficile*, Toxin A/B; *E. coli* O157; Enterotoxigenic *E. coli* (ETEC) LT/ST; Shiga-like toxin-producing *E. coli* (STEC) stx1/stx2; *Salmonella*; *Shigella*; *Vibrio cholerae*; *Yersinia enterocolitica*; adenovirus 40/41; norovirus GI/GII; rotavirus A; *Giardia*; *Cryptosporidium*; and *Entamoeba histolytica*. The GPP has been previously validated against standard clinical assays for the detection of enteric pathogens in multiple countries with >90% sensitivity and ≥99% specificity across pathogens (e.g. [24]). We performed the GPP protocol per manufacturer's instructions: briefly, we pretreated specimens with 1 mL of ASL stool lysis buffer (Qiagen, Hilden, Germany) and co-extracted DNA and RNA using the QIAcube HT platform and the QIAamp 96 Virus QIAcube HT Kit (Qiagen, Hilden, Germany). We assayed 11% of samples in duplicate. Per GPP protocol, all samples included an internal inhibition control (MS2). Additionally, each assay run included at least one negative extraction control (ASL buffer only), sample process control (ASL buffer+MS2) and no-template PCR control. We analysed all extracted DNA and RNA within 24 h of extraction. More detail on methods can be also found in previously-described methods of the Maputo Sanitation Trial [10].

### Anthropometry metrics

Pairs of trained anthropometrists measured the height and weight of each child during the sixth round of follow-up, concurrent with stool specimen collection, using protocols from the Anthropometric Indicators Measurement Guide by the Food and Nutrition Technical Assistance (FANTA) project [25]. Anthropometrists measured child height (length) to the nearest 0.1 cm with a Seca Infant/Child ShorrBoard (Weigh and Measure, LLC, Olney, MD, USA) and weight to the nearest 0.1 kg via a Seca 876 Mother/Baby scale (Weigh and Measure, LLC). All heights and weights were measured in triplicate, with the median used for analysis. We calculated height-for-age *Z*-scores (HAZ scores), weight-for-age *Z*-scores (WAZ scores) and weight-for-height *Z*-scores (WHZ scores) and associated undernutrition outcomes (stunting, underweight and wasting, respectively) using standard WHO criteria (below −2 standard deviations (s.d.) for the associated indicator).

### Survey data collection

We measured caregiver-reported diarrhoea within the previous 7 days in surveys concurrent to stool collection and anthropometric measurements (round 6) using the locally-defined (‘caregiver-defined’) definition of ‘patla paykana’ and the standard WHO definition of ≥3 loose or watery stools in 24 h, with a visual cue [23].

At baseline and during each round of follow-up, field staff administered surveys that collected information on household demographics; water, sanitation and hygiene conditions; and household socioeconomic status. Because some questions were not included in each round of follow-up, we paired responses to these survey questions with an enteric pathogen, anthropometry and diarrhoea (all collected in round 6) by nearest prior round. For example, if a question was administered in rounds 3 and 5, we selected the response from round 5 in order to match the results from round 6 most closely in time.

### Statistical analysis

We assessed descriptive statistics, including pathogen carriage, diarrhoea, anthropometry and demographic data by age: children <2 years old (within the window where growth trajectories are set [11, 26]) *vs.* children 2 to <5 years old. We used R version 3.4.3 (R Foundation for Statistical Computing, Vienna, Austria [27]), with the *lme4* package for (hierarchical) mixed-effects regression modelling [28], for subsequent statistical analyses. We used mixed-effects Poisson regression to estimate prevalence ratios and associated confidence intervals for stunting, wasting and underweight status, as well as caregiver-reported diarrhoea, by the pathogen detected and included random effects for the compound and household of the study child. We used mixed-effects linear regression to estimate the differences in HAZ, WAZ and WHZ by the pathogen detected and included the same random effects. Consistent with the preregistered analysis plan of the parent study, our adjusted analyses included *a priori* confounders describing sociodemographic differences of the study child and household, as well as the household's placement in the randomised-controlled trial. These factors included treatment group in the parent study, reported water treatment at the household, respondent's highest level of education achieved, age and sex of the child, and household income. For pathogens with significant associations with nutritional outcomes in adjusted models, we then compared the prevalence of pathogen detection between children <2 *vs.* ≥2 years old. For pathogens with <5% prevalence, we only ran unadjusted models to avoid problems with model convergence. We used an *α* of 0.05 to determine statistical significance.

## Results

### Demographics and health of study children

Of 1036 children enrolled in the trial at baseline, 57% were lost to follow-up due to ageing out of the study (20%) or other reasons (37%, e.g. moving out of the study compound); loss to follow-up did not differ by intervention or control group [23]. The remaining 43% of children, combined with new enrolees in subsequent rounds, yielded 516 children in round 6 from whom the stool specimens were collected, tested for enteric pathogens and linked to other demographic variables, diarrhoea and anthropometry outcomes.

Study households varied in income and education of the respondent (generally the mother), and had between 2 and 13 people (mean 3.5) living in each room (Table 1). Study children varied by age (range 12 days–60 months) and sex (49% female). About 15% had siblings <5 years old. Most households obtained their drinking water from a tap in their compound (97%). Few (17%) reported treating their water despite approximately half of the participants (47%) being enrolled in the intervention arm, which included no-cost chlorine treatment at the point-of-collection. Most of those who reported treating their drinking water reported boiling (83/88). Most households had a pour-flush toilet (98%), although >70% of households shared their toilet with at least one other household and 30% shared with >5 households.

**Table 1.** Demographics of study children, Bangladesh, 2016 (*n* = 516)

Characteristic	Mean (s.d.) or *N* (%)
Mother's education	
Beyond secondary school	37 (7.2%)
Completed secondary school	26 (5.0%)
Some secondary school	190 (37%)
Completed primary school	67 (13%)
Some primary school	147 (29%)
No formal school	49 (9.5%)
Income (BDT^a^)	17 118 (18 879)
Age of child (months)	31.9 (15.8)
Female child	253 (49%)
Number of people per room in household	3.5 (1.3)
Number of under-5 children per room in household	0.9 (0.4)
Number of people in household	4.8 (1.8)
Number of under-5 children in household	
1	440 (85%)
2	70 (14%)
3	6 (1.2%)
Main water source	
Tap in compound	498 (97%)
Tap in public location	17 (3.3%)
Tap in neighbour's compound	1 (0.2%)
Any reported water treatment	88 (17%)
Any boiling	83 (16%)
Any use of ceramic filter	8 (1.6%)
Any use of cloth filter reported	38 (7.4%)
Intervention arm	244 (47%)
Toilet within compound	
Flush/pour flush connected to tank or pit	497 (96%)
Flush/pour flush connected to sewer	8 (1.6%)
Pit latrine	7 (1.4%)
No toilet	4 (0.8%)
Number of households sharing toilet	
0 (no sharing)	150 (29%)
1	81 (16%)
2	42 (8.1%)
3	43 (8.3%)
4	21 (4.1%)
5	23 (4.5%)
>5	156 (30%)

a Bangladesh Taka.

Few children (6%) had diarrhoea in the week preceding the interview, as reported by their caregiver, but most (88%) had at least one enteric pathogen in the stool specimen collected (Table 2). The most frequently detected pathogens were *Giardia* spp. (40% prevalence), *Salmonella enterica* (33%), enterotoxigenic *E. coli* (ETEC, 28%), *Shigella* spp. (27%), *Campylobacter* spp. (20%) and norovirus (16%). We detected *Giardia* spp. and *Shigella* spp. more often in the specimens from older children (≥2 years old) compared with younger children, while we detected *S. enterica* more often in the stool of younger children. Mean HAZ (−1.32), WAZ (−1.19) and WHZ (−0.64) were all negative, indicating many children were undernourished. About 26% of children were stunted and 24% were underweight, while 7% were wasted. Children ≥2 years old were more likely to be stunted or underweight.

**Table 2.** Diarrhoea, enteric pathogens and malnutrition in study children in Bangladesh, 2016 (*n* = 516)

	Mean (s.d.) or *N* (%)		
Characteristic	All <5 YO	Children <2 YO	Children 2–5 YO
Diarrhoea reported in last 7 days			
Caregiver-defined	29 (5.6%)	13 (7.0%)	16 (4.8%)
WHO-defined	41 (7.9%)	20 (11%)	21 (6.4%)
Pathogens detected in stool			
*Giardia* spp***	204 (40%)	27 (15%)	177 (54%)
Salmonella enterica***	170 (33%)	95 (51%)	75 (23%)
ETEC	143 (28%)	47 (25%)	96 (29%)
*Shigella* spp.***	140 (27%)	28 (15%)	112 (34%)
*Campylobacter* spp.	101 (20%)	45 (24%)	56 (17%)
Norovirus	80 (16%)	33 (18%)	47 (14%)
*E. coli* o157	35 (6.8%)	8 (4.3%)	27 (8.2%)
STEC	29 (5.6%)	8 (4.3%)	21 (6.4%)
*C. difficile*	21 (4.1%)	13 (7.0%)	8 (2.4%)
*Cryptosporidium*	19 (3.7%)	12 (6.5%)	7 (2.1%)
Adenovirus 40/41	13 (2.5%)	12 (6.5%)	1 (0.3%)
Rotavirus	4 (0.8%)	3 (1.6%)	1 (0.3%)
*E. histolytica*	3 (0.6%)	0	3 (0.9%)
*V. cholerae*	1 (0.2%)	1 (0.5%)	0
Num. pathogens detected/child			
0	61 (12%)	23 (12%)	38 (12%)
1	154 (30%)	56 (30%)	98 (30%)
2	159 (31%)	65 (35%)	94 (28%)
3	91 (18%)	25 (13%)	66 (20%)
4	39 (7.6%)	14 (8%)	25 (8%)
5	10 (1.9%)	3 (0.2%)	7 (2%)
6	2 (0.4%)	0	2 (0.6%)
HAZ (*n* = 481)	−1.32 (1.11)	−1.13 (1.08)	−1.42 (1.11)
Stunted*	126 (26%)	33 (19%)	93 (30%)
WAZ (*n* = 513)	−1.19 (1.06)	−0.93 (1.02)	−1.33 (1.06)
Underweight**	121 (24%)	30 (16%)	91 (28%)
WHZ (*n* = 487)	−0.64 (1.08)	−0.54 (1.14)	−0.39 (5.72)
Wasted	36 (7.4%)	14 (8.0%)	22 (7.0%)

YO, year-olds.

*P < 0.05 for difference of proportions by age group; **P < 0.01 for difference of proportions by age group; ***P < 0.001 for difference of proportions by age group.

### Associations between enteric pathogen detection and nutrition outcomes

We compared enteric pathogens detected in the stool specimens with concurrent undernutrition indicators in unadjusted models and models adjusted for the study treatment group, age and sex of the child, household income, any reported water treatment and the respondent's highest education attained (Tables 3 and 4). Although children with *Giardia* spp. detected in the stool had >50% increased prevalence of stunting and underweight in unadjusted models, these relationships were elevated but not significant in adjusted models (stunting PR 1.39, 95% CI 0.94–2.06; underweight PR 1.23, 95% CI 0.83–1.83; Table 3). Children with adenovirus 40/41 had more than three times higher prevalence of wasting in unadjusted models, but could not be evaluated in adjusted models due to the few (*n* = 13) detections

**Table 3.** Bivariable (unadjusted) and multivariable (adjusted) analyses of anthropometric outcome cut-offs by pathogen detection in the stool, Bangladesh, 2016

	HAZ (stunting)		WAZ (underweight)		WHZ (wasting)	
Pathogen	PR_stunting_ (95% CI)	aPR_stunting_ (95% CI)^a^	PR_underwt_ (95% CI)	aPR_underwt_ (95% CI)^a^	PR_wasting_ (95% CI)	aPR_wasting_ (95% CI)^a^
*Giardia* spp.	**1.56 (1.10–2.21)**	1.39 (0.94–2.06)	**1.55 (1.09–2.22)**	1.23 (0.83–1.83)	0.77 (0.39–1.55)	0.67 (0.32–1.44)
*Salmonella enterica*	0.83 (0.56–1.21)	0.90 (0.60–1.35)	0.71 (0.47–1.06)	0.81 (0.53–1.24)	0.91 (0.45–1.84)	0.87 (0.42–1.82)
ETEC^b^	0.98 (0.66–1.44)	0.93 (0.62–1.37)	1.14 (0.77–1.68)	1.08 (0.73–1.60)	1.43 (0.72–2.82)	1.38 (0.69–2.74)
*Shigella* spp.	1.05 (0.71–1.55)	0.90 (0.60–1.36)	1.11 (0.75–1.64)	0.90 (0.60–1.37)	1.06 (0.51–2.19)	0.97 (0.45–2.08)
*Campylobacter* spp.	1.25 (0.83–1.89)	1.21 (0.79–1.87)	0.76 (0.47–1.24)	0.73 (0.44–1.21)	0.80 (0.33–1.93)	0.73 (0.30–1.79)
Norovirus	1.21 (0.77–1.90)	1.25 (0.79–1.98)	1.29 (0.82–2.03)	1.40 (0.89–2.23)	0.89 (0.34–2.28)	0.91 (0.35–2.35)
*E. coli* o157	0.80 (0.37–1.71)	0.83 (0.38–1.84)	0.97 (0.47–1.98)	0.94 (0.45–1.98)	0.38 (0.05–2.78)	0.32 (0.04–2.39)
STEC^c^	1.03 (0.48–2.21)	0.86 (0.39–1.89)	1.18 (0.58–2.42)	1.09 (0.52–2.28)	–	–
*C. difficile*	0.84 (0.31–2.28)	–	0.84 (0.31–2.28)	–	2.24 (0.69–7.30)	–
*Cryptosporidium*	1.13 (0.46–2.76)	–	0.89 (0.33–2.41)	–	1.53 (0.37–6.38)	–
Adenovirus 40/41	–	–	0.98 (0.31–3.08)	–	**3.31 (1.02–10.8)**	–
Rotavirus	1.27 (0.18–9.12)	–	1.06 (0.15–7.59)	–	–	–
*E. histolytica*	1.27 (0.18–9.12)	–	1.42 (0.20–10.1)	–	4.61 (0.63–33.6)	–
*V. cholerae*	–	–	–	–	–	–

a Adjusted for treatment group, age (months), sex, income, reported water treatment and respondent's highest education; ‘−’ indicates too few positive observations for stable model convergence. As a rule, organisms with <5% prevalence were not analysed in multivariable (adjusted) associations. **Bold** indicates significant associations at *P* < 0.05.

b Enterotoxigenic *E. coli*.

c Shiga-toxin-producing *E. coli*.

**Table 4.** Bivariable (unadjusted) and multivariable (adjusted) analyses of continuous anthropometric outcomes by pathogen detection in the stool, Bangladesh, 2016

	HAZ		WAZ		WHZ	
Pathogen	*β*_stunting_ (95% CI)	Adusted *β*_stunting_ (95% CI)^a^	*β*_underwt_ (95% CI)	Adjusted *β*_underwt_ (95% CI)^a^	*β*_wasting_ (95% CI)	Adjusted *β*_wasting_ (95% CI)^a^
*Giardia* spp.	**−0.36 (−0.56 to −0.16)**	**−0.22 (−0.44 to 0.00)**	**−0.39 (−0.58 to −0.21)**	−0.18 (−0.38 to 0.02)	0.35 (−0.49 to 1.19)	0.43 (−0.49 to 1.36)
*Salmonella enterica*	**0.26 (0.05–0.47)**	0.15 (−0.07 to 0.37)	**0.33 (0.13–0.52)**	0.18 (−0.02 to 0.39)	−0.09 (−0.97, 0.79)	−0.19 (−1.12 to 0.73)
ETEC^b^	−0.01 (−0.23 to 0.21)	0.03 (−0.19 to 0.25)	−0.14 (−0.34 to 0.06)	−0.08 (−0.28 to 0.12)	−0.45 (−1.36 to 0.46)	−0.50 (−1.43 to 0.42)
*Shigella* spp.	0.17 (−0.40 to 0.05)	−0.04 (−0.27 to 0.19)	−0.18 (−0.39 to 0.02)	0.03 (−0.18 to 0.24)	0.72 (−0.21 to 1.65)	0.82 (−0.15 to 1.79)
*Campylobacter* spp.	−0.11 (−0.36 to 0.13)	−0.16 (−0.41 to 0.09)	−0.03 (−0.26 to 0.20)	−0.07 (−0.30 to 0.16)	−0.18 (−1.22 to 0.85)	−0.45 (−1.50 to 0.61)
Norovirus	−0.04 (−0.32 to 0.23)	−0.08 (−0.35 to 0.20)	0.01 (−0.25 to 0.26)	−0.05 (−0.30 to 0.20)	−0.27 (−1.41 to 0.88)	−0.35 (−1.50 to 0.81)
*E. coli* o157	−0.01 (−0.41 to 0.38)	−0.01 (−0.41 to 0.38)	−0.01 (−0.38 to 0.36)	0.03 (−0.33 to 0.40)	−0.07 (−1.69 to 1.55)	−0.44 (−2.09 to 1.21)
STEC^c^	−0.09 (−0.53 to 0.35)	0.00 (−0.43 to 0.44)	−0.17 (−0.57 to 0.22)	−0.14 (−0.53 to 0.25)	−0.23 (−2.03 to 1.57)	−0.28 (−2.12 to 1.56)
*C. difficile*	0.16 (−0.36 to 0.68)	–	0.08 (−0.40 to 0.56)	–	−0.26 (−2.39 to 1.87)	–
*Cryptosporidium*	−0.25 (−0.79 to 0.29)	–	−0.23 (−0.71 to 0.26)	–	−0.15 (−2.34 to 2.03)	–
Adenovirus 40/41	0.48 (−0.13 to 1.09)	–	0.06 (−0.53 to 0.65)	–	−0.53 (−3.08 to 2.03)	–
Rotavirus	−0.23 (−1.49 to 1.03)	–	−0.34 (−1.38 to 0.70)	–	−0.67 (−5.94 to 4.59)	–
*E. histolytica*	0.56 (−0.69 to 1.82)	–	0.29 (−0.90 to 1.49)	–	−0.35 (−5.62 to 4.92)	–
*V. cholerae*	–	–	–	–	–	–

a Adjusted for treatment group, age (months), sex, income, reported water treatment and respondent's highest education; ‘-’ indicates too few positive observations for stable model convergence. As a rule, organisms with <5% prevalence were not analysed in multivariable (adjusted) associations. **Bold** indicates significant associations at *P* < 0.05.

b Enterotoxigenic *E. coli*.

c Shiga-toxin-producing *E. coli*.

When examining HAZ, WAZ and WHZ scores continuously in adjusted models, children with *Giardia* spp. had −0.22 s.d. lower HAZ (95% CI −0.44 to 0.00) (Table 4). Although children with *Giardia* spp. detected had lower WAZ while children with *Salmonella* spp. detected had higher HAZ and WAZ in unadjusted models, these relationships were attenuated in adjusted models.

**Fig. 1.**
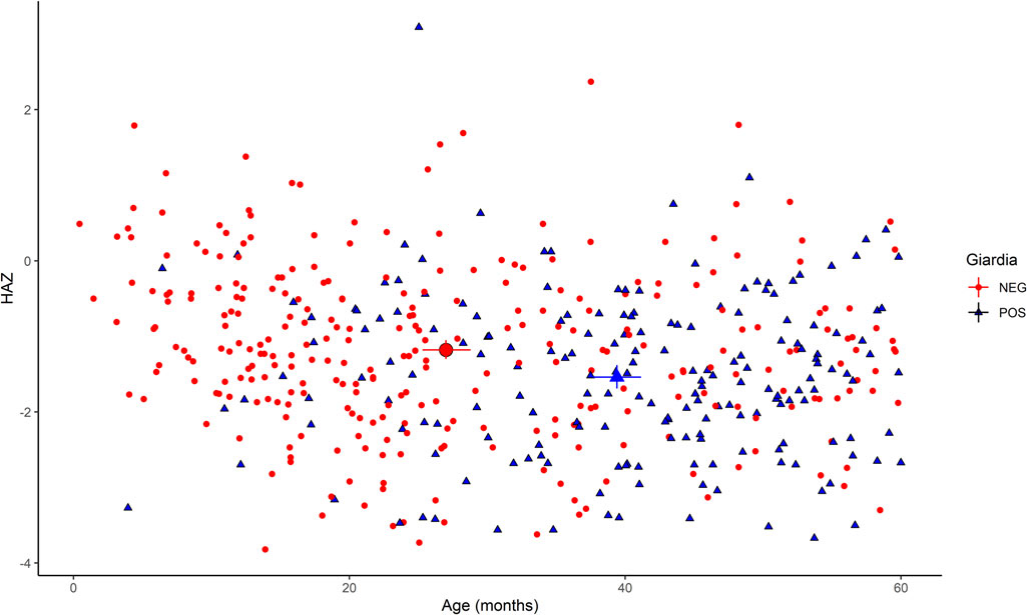
HAZ scores by age and *Giardia* spp. detection. Mean age and HAZ for children (*n* = 516) with and without *Giardia* spp. detection in the stool is denoted by large blue triangles and red circles, respectively, with 95% confidence intervals for the *X*- and *Y*-axis means denoted by lines.

In a subsequent analysis of *Giardia* spp. detection by age (children <2 *vs.* ≥2 years old), *Giardia* spp. was more likely to be detected in children ≥2 years old (Fig. 1): adjusted PR 3.67, 95% CI 2.43–5.52 (Table S1). In adjusted models, *Shigella* spp. was also more likely to be detected in children ≥2 years old (PR 2.19, 95% CI 1.44–3.34) while *S. enterica* (PR 0.42, 95% CI 0.31–0.58) and *Campylobacter* spp. (PR 0.64, 95% CI 0.43–0.95) were less likely to be detected in ≥2 years old, as compared with <2 years old (Table S1).

### Associations between enteric pathogen detection and reported diarrhoea

We compared enteric pathogen detection in the stool to caregiver-defined and WHO-defined diarrhoea in the previous week in both unadjusted and adjusted models using previously-described covariates (Table 5). No pathogens were significantly associated with diarrhoea, using either definition, in adjusted models. In unadjusted models, adenovirus 40/41 detection was significantly associated with caregiver-defined diarrhoea (PR 5.31, 95% CI 1.43–19.6) and rotavirus detection was significantly associated with WHO-defined diarrhoea (PR 6.35, 95% CI 1.44–28.0), though each were detected in very small proportions of children's stool (2.5% and 0.8%, respectively). Prevalence of reported diarrhoea, using either definition, did not vary significantly with the number of pathogens detected in the stool.

**Table 5.** Prevalence and prevalence ratios (PR) of diarrhoea by pathogen detection in the stool, Bangladesh, 2016

Pathogen	Prevalence of diarrhoea in positive detects	Prevalence of diarrhoea in non-detects	PR_diarrhoea_ (95% CI)	aPR_diarrhoea_ (95% CI)^a^
	*Caregiver-defined*^b^	*Caregiver-defined*^b^	*Caregiver-defined^b^*	*Caregiver-defined^b^*
	WHO-defined^c^	WHO-defined^c^	WHO-defined^c^	WHO-defined^c^
*Giardia* spp.	*9/204 (4.4%)*	*20/312 (6.4%)*	*0.63 (0.28, 1.42)*	*0.82 (0.32, 2.13)*
	13/204 (6.4%)	28/312 (9.0%)	0.70 (0.36, 1.37)	0.86 (0.42, 1.76)
*Salmonella enterica*	*12/170 (7.1%)*	*17/349 (4.9%)*	*1.45 (0.68, 3.09)*	*1.32 (0.59, 2.95)*
	14/170 (8.2%)	27/346 (7.8%)	1.05 (0.55, 2.01)	0.91 (0.46, 1.81)
ETEC^d^	*13/143 (9.1%)*	*16/373 (4.3%)*	*2.09 (0.98, 4.43)*	*2.05 (0.95, 4.43)*
	13/143 (9.1%)	28/373 (7.5%)	1.20 (0.62, 2.33)	1.09 (0.55, 2.15)
*Shigella* spp.	*8/140 (5.7%)*	*21/376 (5.6%)*	*1.03 (0.44, 2.40)*	*1.28 (0.50, 3.28)*
	14/140 (10%)	27/376 (7.2%)	1.36 (0.71, 2.62)	1.78 (0.90, 3.53)
*Campylobacter* spp.	*6/101 (5.9%)*	*23/415 (5.5%)*	*1.03 (0.41, 2.57)*	*0.76 (0.27, 2.16)*
	8/101 (7.9%)	33/415 (8.0%)	0.99 (0.45, 2.15)	0.68 (0.30, 1.56)
Norovirus	*4/80 (5.0%)*	*25/436 (5.7%)*	*0.89 (0.30, 2.61)*	*0.77 (0.23, 2.54)*
	7/80 (8.8%)	34/436 (7.8%)	1.13 (0.50, 2.57)	0.97 (0.41, 2.29)
*E. coli* o157	*1/35 (2.9%)*	*28/481 (5.8%)*	*0.57 (0.07, 4.54)*	*0.55 (0.06, 5.32)*
	2/35 (5.7%)	39/481 (8.1%)	0.73 (0.17, 3.08)	0.81 (0.19, 3.51)
STEC^e^	*0/29 (0.0%)*	*29/487 (6.0%)*	*–*	*–*
	1/29 (3.4%)	40/487 (8.2%)		
*C. difficile*	*2/21 (9.5%)*	*27/495 (5.5%)*	*2.34 (0.50, 10.9)*	*–*
	1/21 (4.8%)	40/495 (8.1%)	0.63 (0.08, 4.62)	
*Cryptosporidium*	*3/19 (16%)*	*26/497 (5.2%)*	*2.51 (0.70, 8.97)*	*–*
	2/19 (11%)	39/497 (7.8%)	1.35 (0.32, 5.66)	
Adenovirus 40/41	***3/13 (23%)***	***26/503 (5.2%)***	***5.31 (1.43, 19.6)***	*–*
	3/13 (23%)	38/503 (7.6%)	3.04 (0.90, 10.2)	
Rotavirus	*1/4 (25%)*	*28/512 (5.5%)*	*3.57 (0.43, 29.7)*	*–*
	**2/4 (50%)**	**39/512 (7.6%)**	**6.35 (1.44, 28.0)**	
*E. histolytica*	*0/3 (0.0%)*	*29/513 (5.7%)*	*–*	*–*
	0/3 (0.0%)	41/513 (8.0%)		
*V. cholera*	*1/1 (100%)*	*28/515 (5.4%)*	*–*	*–*
	1/1 (100%)	40/515 (7.8%)		
None detected	*4/61 (6.6%)*	*25/455 (5.5%)*	*–*	*–*
	5/61 (8.2%)	36/455 (7.9%)		
Number of detects				
Continuous			*1.27 (0.91, 1.78)*	*1.18 (0.87, 1.59)*
			1.08 (0.83, 1.41)	1.05 (0.82, 1.35)
Any co-detection	*20/301 (6.6%)*		*1.05 (0.34, 3.26)*	*1.04 (0.32, 3.45)*
	26/301 (8.6%)		1.07 (0.41, 2.82)	1.10 (0.42, 2.94)
Single detection	*5/154 (3.2%)*		*0.46 (0.12, 1.75)*	*0.50 (0.11, 2.19)*
	10/154 (6.5%)		0.80 (0.27, 2.35)	0.88 (0.29, 2.64)
No detection	*4/61 (6.6%)*		*Ref.*	*Ref.*
	5/61 (8.2%)		Ref.	Ref.

a Adjusted for treatment group, age (months), water treatment, sex, income and respondent's highest education.

b *n* = 29 reported.

c *n* = 41 reported.

d Enterotoxigenic *E. coli*.

e Shiga-toxin-producing *E. coli*. ‘−’ indicates too few positive observations for stable model convergence. As a rule, organisms with <5% prevalence were not analysed in multivariable (adjusted) associations. **Bold** indicates significant associations at *P* < 0.05.

## Discussion

This study assessed enteric pathogen carriage and nutritional outcomes in a cross-section of children <5 years old enrolled in an existing trial cohort from urban Dhaka, Bangladesh [23]. We observed a high prevalence of enteric pathogens detected in the stool (88%) despite the low caregiver-reported prevalence of diarrhoea (6–8%, depending on definition). Although rarely detected, viruses were associated with caregiver-reported and/or WHO-defined diarrhoea, but not other pathogens. Children with *Giardia* spp. detected in the stool had significantly lower HAZ scores and were somewhat more likely to be stunted (though this difference was not statistically significant); however *Giardia* spp. detections were more likely in children older than 24 months (outside the period when growth trajectories may be altered [11, 26, 29]).

Our results contribute to the growing body of evidence that exposure to or infection with enteric pathogen – as measured by molecular assays of the stool – may be high despite the low prevalence of reported diarrhoea. Similar results have been found in other low-income, urban settings in children under 5 [10] and multisite studies of urban and rural settings focusing on children in the first 2 years of life [9]. Taken together, these findings represent a new challenge for implementers of changes to environmental infrastructure and systems, such as WASH, to mitigate enteric pathogen exposures (measured via the stool detection of pathogens, a more sensitive metric) as contrasted with previous studies of diarrhoea or clinical illness. However, enteric pathogen detection may also be more useful as an outcome in randomised-controlled trials given the higher prevalence compared with diarrhoea [30, 31] and findings that reported diarrhoea may be associated with primarily viral pathogens (generally not effectively mitigated by water and sanitation systems [10, 32]). Thus, the use of caregiver-reported paediatric diarrhoea alone as an outcome measure may be susceptible to biasing trial results towards the null [32].

Our findings are consistent with other results suggesting *Giardia* spp. as a highly prevalent, often asymptomatic pathogen associated with growth faltering in children in low-income settings [6, 8, 12, 33]. However, our evidence does not suggest that *Giardia* spp. was a causative agent for child stunting, since *Giardia* spp. was detected more often in children older than 2 years of age. Instead, our analysis suggests these already undernourished children may instead be more likely to be infected with or unable to clear *Giardia* spp. infections, possibly due to their malnutrition [34, 35], though we cannot characterise the directionality of this association. Disproportionately higher rates of *Giardia* spp. infections in older children may be explained by a combination of weaning and increased mobility, both of which increase potential exposure rates, since *Giardia* spp. infection is rarely observed in very young, less-mobile, and often breastfed infants and infection rates increase with age [33]. Alternatively, one may hypothesise that children in our study had ‘chronic’ *Giardia* spp. infections (>6 months in some cases [36]) resulting in ongoing shedding due to previous or repeat asymptomatic infections and been unlikely to receive anti-protozoal drugs due to the absence of symptoms.

In contrast to our findings, evidence from other studies still strongly supports *Giardia* spp. as a key infection that drives reduced growth in young children worldwide, though with mixed evidence on the mechanism of action (e.g. inflammatory pathways, increased gut permeability or other morphological changes characteristic of EED [5]). Longitudinal data from the Etiology, Risk Factors, and Interactions of Enteric Infections and Malnutrition and Consequences for Child Health and Development Project (MAL-ED study) indicated *Giardia* spp. infection in the first 6 months of life was associated with decreased HAZ at 24 months of age [4, 33]. Mouse models have shown persistent *Giardia* spp. infection to be associated with increased inflammatory response, changes to gut villi structure and poorer growth [37], and there is evidence that adults in high-income settings have experienced changes to gut morphology following a *Giardia* spp. outbreak [36].

There are important limitations to consider in this analysis. Although enteric infections may be persistent in young children, the stool specimens were analysed using molecular detection methods that detected the presence/absence at a single surveillance point; intermittently shed pathogens may be missed in a single stool analysis, which could have been avoided if multiple stool specimens were analysed. Among those specimens with positive detects, we cannot identify whether and when a clinical infection was present (and/or duration), including its intensity, or whether shedding of a pathogen was due to other causes [38]. Additionally, although WASH conditions were consistent within the study population and we adjusted for water treatment, income and respondent's education among other potential confounders, other environmental, socioeconomic and nutritional variables that we did not measure could have impacted pathways we assessed. We matched survey data to stool collections as closely as possible in time with outcome ascertainment; however, assumptions about the temporal stability of environmental conditions and sociodemographic characteristics (e.g. consistent conditions across time) were necessary that could have affected the exposure–infection relationships. Cross-sectional analyses have strict temporal constraints and directionality of association cannot be directly assessed.

Enteric infections, as detected via pathogen carriage in the stool, are highly prevalent in low-income children despite the low concurrent prevalence of reported diarrhoea. *Giardia* spp. is the most common parasitic infection in young children in many low-income settings and is generally not associated with clinical diarrhoea, but is associated with growth faltering. Results from this analysis contribute to the growing literature surrounding associations between *Giardia* spp. infections and malnutrition and linear growth in these settings [6, 33, 36, 37, 39, 40], yet may suggest *Giardia* spp. infections as a symptom, rather than a cause, of undernutrition. Because *Giardia* and other enteric pathogens detected in the stool are unambiguous markers of past exposure and because they are on the causal pathway to infection, disease and longer-term health outcomes, such targets may offer potential advantages as outcomes in trials of environmental improvement (e.g. WASH) interventions.

## Supplementary material

For supplementary material accompanying this paper visit https://doi.org/10.1017/S0950268820000369. click here to view supplementary material
