# Supplementary material for: Associations between enteric pathogen carriage and height-for-age, weight-for-age and weight-for-height in children under 5 years old in urban Dhaka, Bangladesh
Source: Epidemiol Infect. 2020 Feb 27;148:e39. doi: 10.1017/S0950268820000369 (PMC7058651; doi:10.1017/S0950268820000369)
Supplement: Supplementary file 1 [file S0950268820000369sup001.docx]

| Table S1: Bivariable (unadjusted) and multivariable (adjusted) analyses of pathogen detection by age: 2-5 years old vs. <2 years old (reference group) | | |
| --- | --- | --- |
| Pathogen | Unadjusted PR (95% CI) | Adjusted PR (95% CI)^1^ |
| *Giardia* spp. | **3.71 (2.47, 5.56)** | **3.67 (2.43, 5.52)** |
| *Salmonella* *enterica* | **0.44 (0.33, 0.60)** | **0.42 (0.31, 0.58)** |
| ETEC^2^ | 1.15 (0.81, 1.63) | 1.14 (0.80, 1.63) |
| *Shigella* spp. | **2.25 (1.49, 3.41)** | **2.19 (1.44, 3.34)** |
| *Campylobacter* spp. | 0.70 (0.47, 1.04) | **0.64 (0.43, 0.95)** |
| Norovirus | 0.80 (0.51, 1.25) | 0.80 (0.51, 1.26) |
| *E. coli* o157 | 1.75 (0.78, 3.92) | 1.56 (0.65, 3.76) |
| STEC^3^ | 1.46 (0.65, 3.30) | 1.28 (0.56, 2.93) |
| *C. difficile* | **0.28 (0.11, 0.72)** | - |
| *Cryptosporidium* | **0.33 (0.13, 0.84)** | - |
| Adenovirus 40/41 | **0.06 (0.01, 0.46)** | - |
| Rotavirus | 0.20 (0.02, 1.92) | - |
| *E. histolytica* | - | - |
| *V. cholerae* | - | - |

^1^Adjusted for treatment group, sex, income, reported water treatment, and respondent’s highest education; “-“ indicates too few positive observations for stable model convergence. As a rule, organisms with <5% prevalence were not analyzed in multivariable (adjusted) associations. **Bold** indicates significant associations at p<0.05. ^2^Enterotoxigenic *E. coli*. ^3^Shiga-toxin-producing *E. coli*.
